# Supplementary material for: Divergent effects of climate change on future groundwater availability in key mid-latitude aquifers
Source: Nat Commun. 2020 Jul 24;11:3710. doi: 10.1038/s41467-020-17581-y (PMC7382464; doi:10.1038/s41467-020-17581-y)
Supplement: Supplementary file 1 — Supplementary Information [file 41467_2020_17581_MOESM1_ESM.pdf]

Supplementary Information

Divergent effects of climate change on future groundwater availability in key mid-latitude aquifers

Authors: Wen-Ying Wu[1,2], Min-Hui Lo[1*], Yoshihide Wada[3], James S. Famiglietti[4], John T. Reager[5], Pat J.-F. Yeh[6], Agnès Ducharne[7], Zong-Liang Yang[2]

[1]Department of Atmospheric Sciences, National Taiwan University, Taipei 10617, Taiwan

[2]Department of Geological Sciences, The University of Texas at Austin, Austin, Texas 78712, USA

[3]International Institute of Applied Systems Analysis, Laxenburg, Austria

[4]School of Environment and Sustainability and Global Institute for Water Security, University of Saskatchewan, Saskatoon, Canada

[5]NASA Jet Propulsion Laboratory, California Institute of Technology, Pasadena, California 91109, USA

[6]School of Engineering, Monash University Malaysia, Malaysia

[7]Sorbonne Universités, UPMC, CNRS, EPHE, UMR 7619 METIS, 4 place Jussieu, 75005, Paris, France

[*] Corresponding author: Min-Hui Lo: minhuilo@ntu.edu.tw

Supplementary Information:

Supplementary Discussion

Supplementary Figure 1-21

Supplementary Table 1-3

References

Supplementary Discussion

Comparison to the observations (GRACE) and models (CESM-LE and CMIP5)

A Taylor diagram provides a statistical summary of how well patterns match and is widely used to compare the outputs from models with observations[1]. Supplementary Figure 1 shows a comparison of the globally averaged seasonal time series (climatology for 1980–2005) from models and the GRACE observations (climatology for 2003–2012). First, the correlation coefficients for the radial line denote the relationship between GRACE and the models, indicating similarities on seasonal time scales. Second, the normalized standard deviations (standard deviation of the model divided by that of the observations) on the x- and y-axes indicate if the variance of the model is larger or smaller than those in the observations. The points inside the dashed curved line mean that the model simulations have lower variability than the GRACE observations. By contrast, the models with normalized standard deviation are higher than one has higher variability than the GRACE observations. For example, GISS observations have more than two times the standard deviation of the GRACE observations because of a larger seasonal cycle. The reasons for the biases in the model could be from simulated atmospheric forcing from the configurations, such as soil type, layers, and depths, or parameterizations in different land surface models. On combining the correlation and standard deviation, the points closer to the reference point on the x-axis show a better agreement, making the ensemble mean of the CESM-LE one of the realistic models representing terrestrial water storage. Supplementary Figure 1 shows that the CESM agrees with observations for the simulated seasonal cycle of total water storage.

Other potential factors for climate-driven groundwater storage changes

In this study, we conclude that rainfall, ET, and snowmelt are dominant factors for groundwater changes. Here we discuss some other potential factors. Changes in frozen soil, the frequency, and intensity of precipitation, and land use can also have impacts on groundwater changes. Supplementary Figure 12-18 show the preliminary results of these factors over seven studied regions.

Results show that there are declines of soil ice fraction (fractions of ice to ice+liquid in soil). The infiltration rate of snowmelt into the soil column is sensitive to the ice fraction in the soil. Under climate change, extreme precipitation is expected. We analyze the probability distribution of precipitation in the wettest month among 30 years and 30 ensemble simulations. At the end of the century, the probability distributions shift to a larger amount (shift to right-side in Supplementary Figure 12-18e), or the standard deviation increases.

In some regions, groundwater storage relies on extreme rainfall[2]. We already include impacts of average precipitation to groundwater in the main text. However, to examine how extreme precipitation can affect groundwater storage through changes in soil macropores and fractures needs further studies.

A transient land cover is one of the input data of CESM, which is prescribed (in Supplementary Figure 12-18f). In the future, the C3 crop of agriculture is projected to increases, while other natural vegetation types are projected to decline relatively over seven studies regions. Changes in land cover can change the terrestrial water cycle with human water usage or different surface

properties. Further sensitivity tests are required to isolate the impacts of different land cover to groundwater.

Description of dynamical vegetation model:

In the simulations that we used, dynamic vegetation models are linked to complex biogeochemical and hydrological responses[3,4] With increasing CO_2 concentrations, transpiration may decrease because of reduced stomatal opening[5,6]. In coupled climate model experiments, other factors, such as temperature, humidity, surface net radiation, soil moisture availability, and vegetation type, can all affect others such as length of the growing season, leaf area index, and stomatal conductance[7]. Hence, the changes in transpiration are not only caused by a single factor but also by complex interactions within the climate system, which then affect the water balance and aquifer recharge rates.

Robustness in groundwater trend due to climate change:

In this study, we estimate the climate-driven groundwater trend with model simulations. Our analysis (Supplementary figure 19) shows that the trend is mostly induced by warming, not by the spin-up process, climate drift, or internal variability. We compared a simulation of pre-industrial with another simulation of historical and then RCP8.5 for 250 years. Two simulations are started with the same initial value. Within the same length of the period, the evolutions of groundwater storage are different, which indicate the impacts of warming by greenhouse gas concentration. Also, the time series from the pre-industrial simulation is stable and has no apparent trend compared to one from historical+RCP8.5, which we use in this study.

Probability of groundwater trend due to climate change:

To assess the uncertainties of internal climate variability in future projections, we further analyzed the uncertainties through the spreads among the simulations of 30 ensemble members in CESM-LE. Results show that the ranges of simulated groundwater storage changes among ensemble members vary by different aquifers. (Supplementary Figure 21). The ensemble consensus on the sign of changes (+ or –) is all larger than 67% for all seven aquifers considered, indicating the robustness of the ensemble averages. For example, all 30 members simulated the declines (increases) in groundwater storage over the Southern Plains and the Middle East (Guarani). In addition, 20, 25, 28, and 29 members simulated increasing groundwater storage over Central Valley, northwestern India, Canning Basin, and North China Plain, respectively.

Comparison with previous studies:

Previous studies of groundwater changes under climate change are based on offline hydrological models or regional groundwater models. Here we compare our results of groundwater recharge changes with the findings in previous studies. Increasing groundwater recharge in Northwestern India and the North China Plain are consistent with results from global hydrological models[10,11]. In the same studies[10,11], they also found groundwater recharges in Southern Plains decreases with climate change. Less groundwater recharge in Southern Plains is also suggested by research using regional groundwater models[12]. The study also concluded that groundwater recharge projections Central Valley has large uncertainties[12].

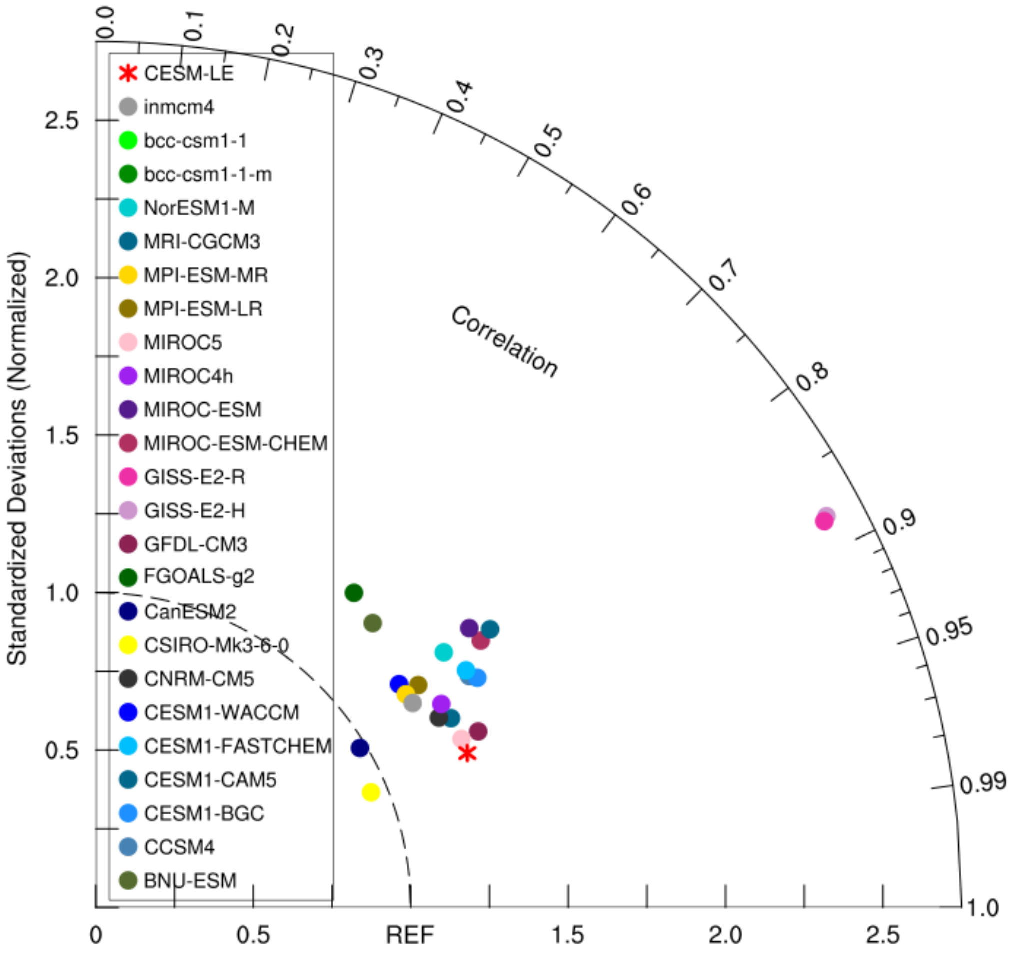

Supplementary Figure 1 | Normalized Taylor diagram presenting a comparison of the GRACE observations (climatology for 2003–2012) with simulations from CMIP5 and CESM-LE (climatology for 1980–2005) for the annual cycle time series of global land water storage. The diagram shows the correlation and ratio of the standard deviation.

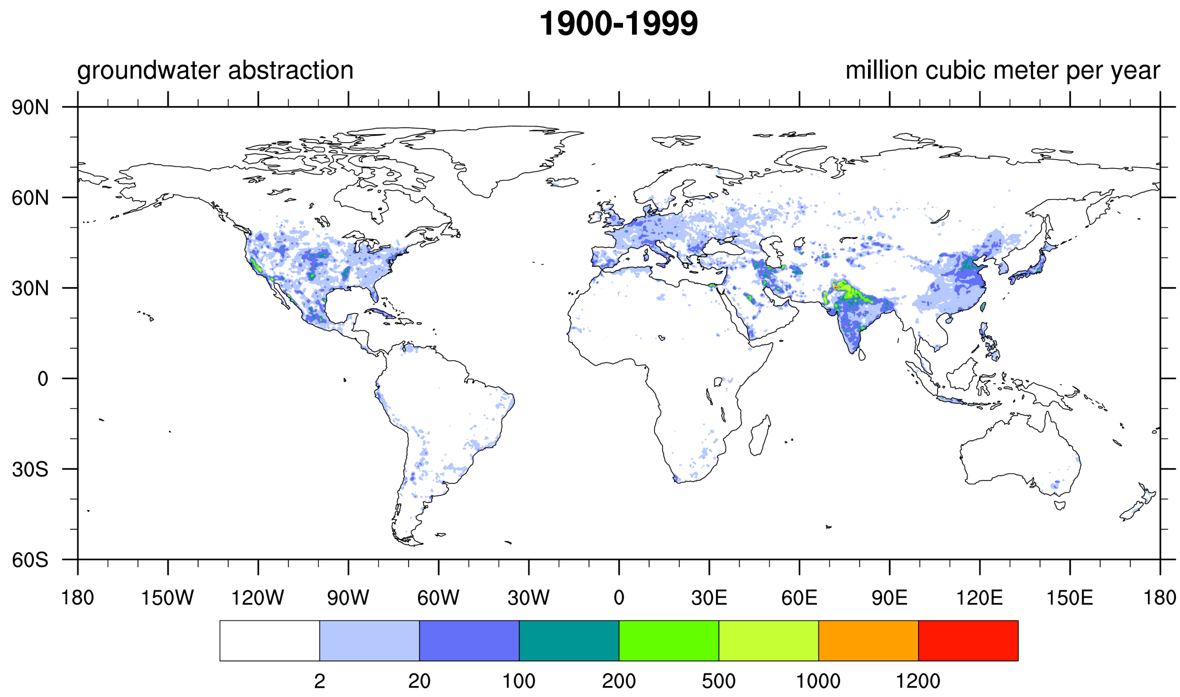

Supplementary Figure 2 | Annual average of groundwater abstraction rate (m^3 yr^{-1}) updated from Wada, 2010[8] that was used in CESM simulation[9].

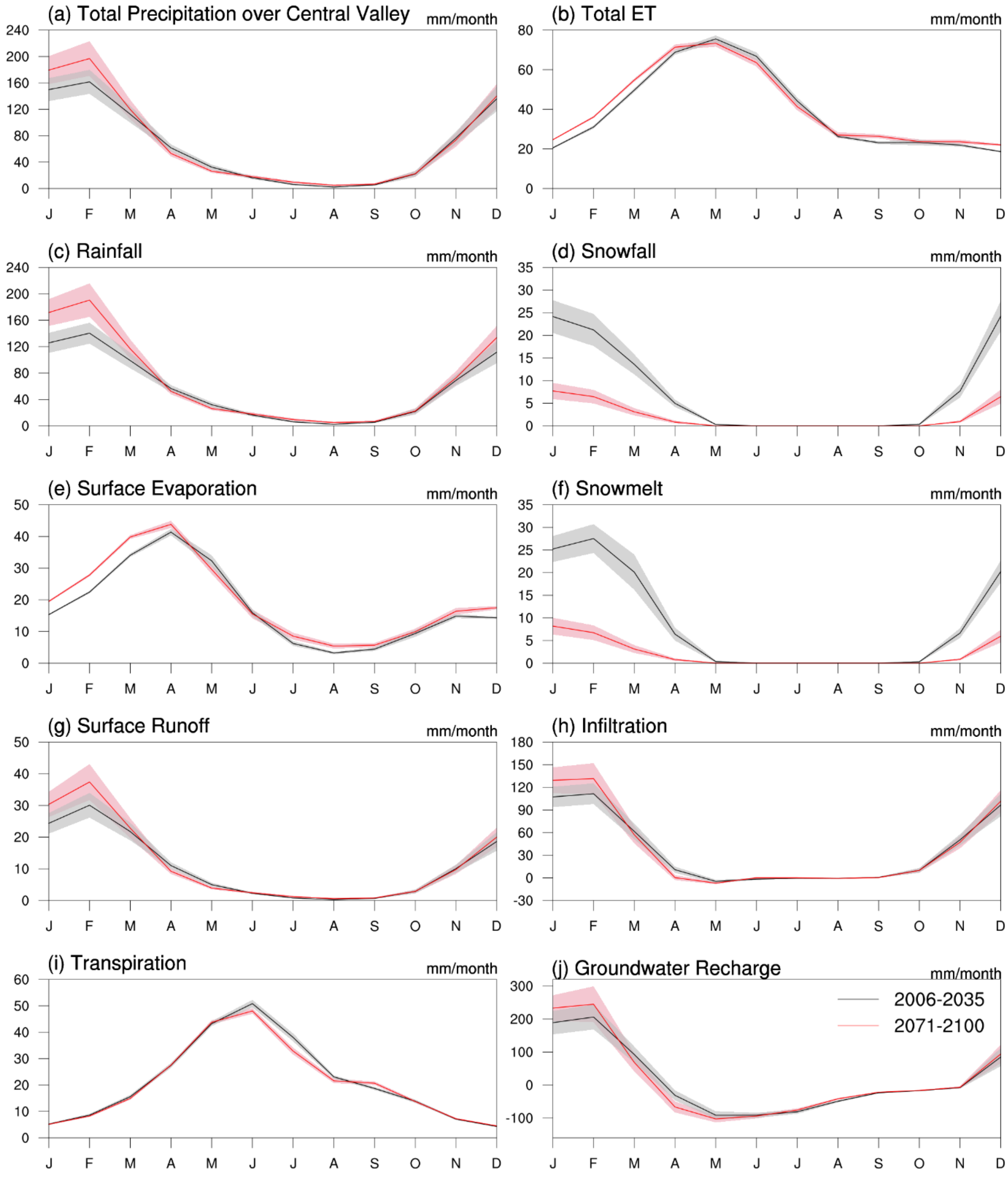

Supplementary Figure 3 | Seasonal cycle of variables over the Central Valley. (a)-(j) Black and red lines represent the ensemble mean during 2006–2035 and 2071–2100, respectively. Shaded areas represent a single standard deviation.

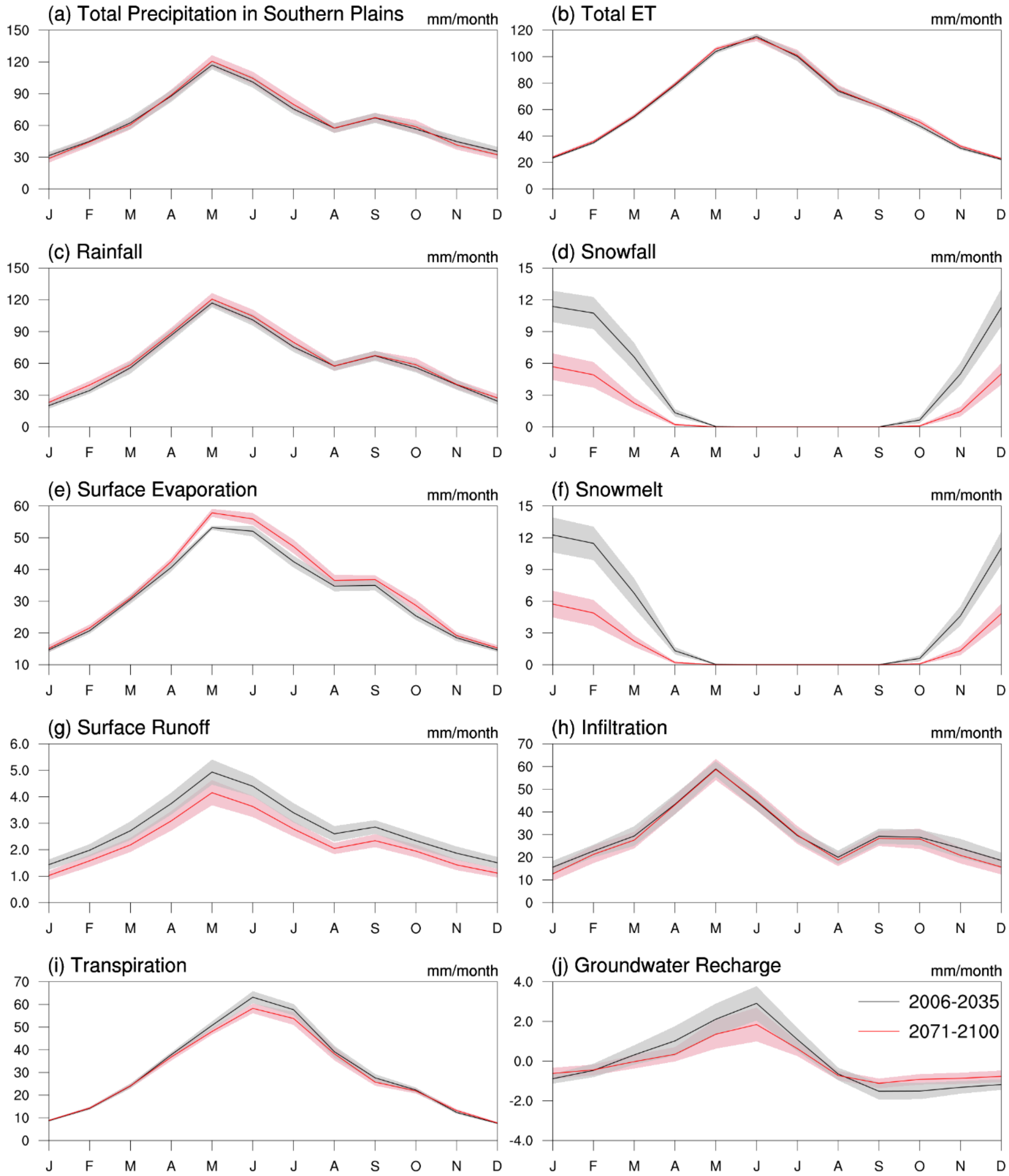

Supplementary Figure 4 | Seasonal cycle of variables over the Southern Plains. (a)-(j) Black and red lines represent the ensemble mean during 2006−2035 and 2071−2100, respectively. Shaded areas represent a single standard deviation.

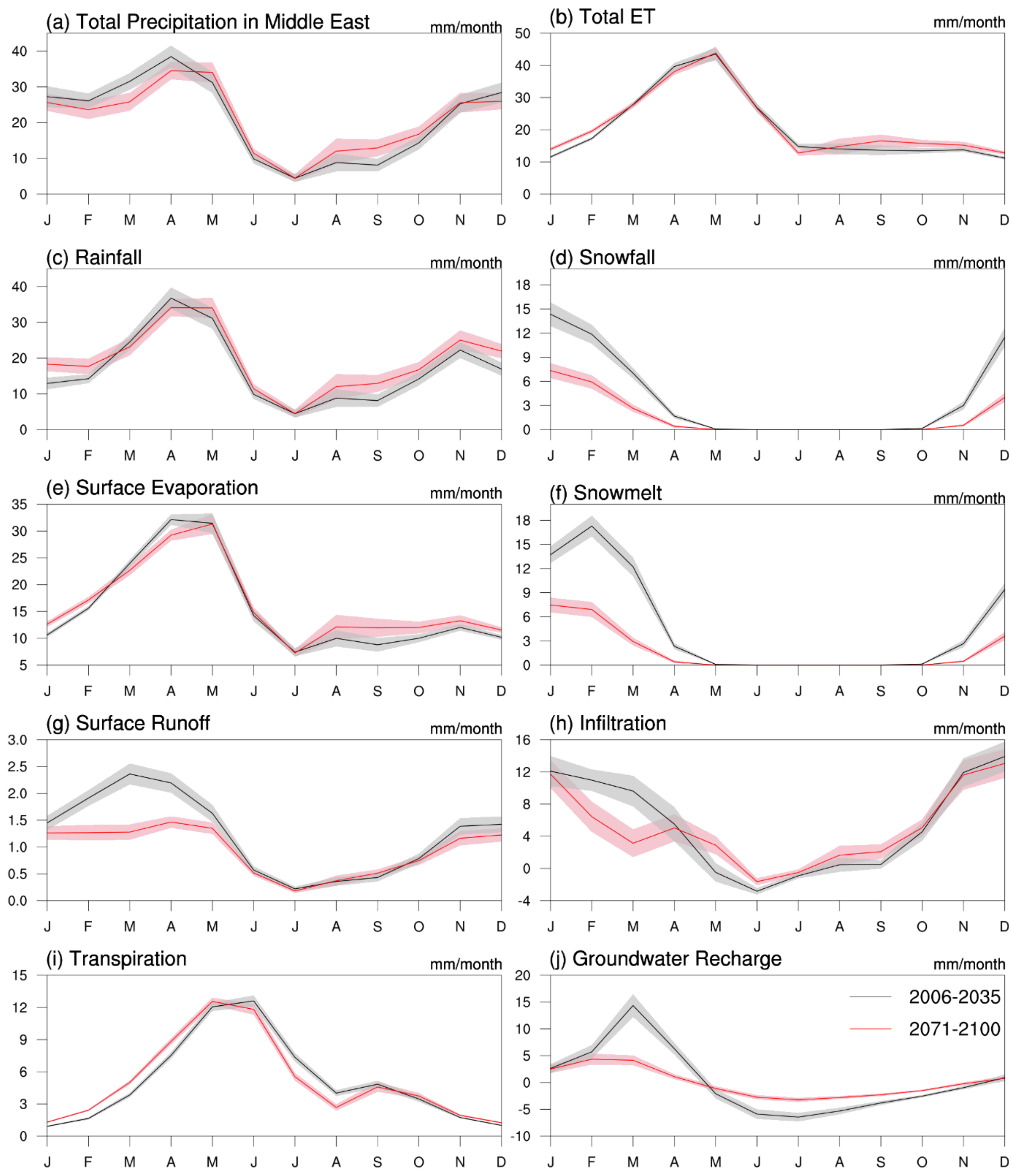

Supplementary Figure 5 | Seasonal cycle of variables over the Middle East. (a)-(j) Black and red lines represent the ensemble mean during 2006–2035 and 2071–2100, respectively. Shaded areas represent a single standard deviation.

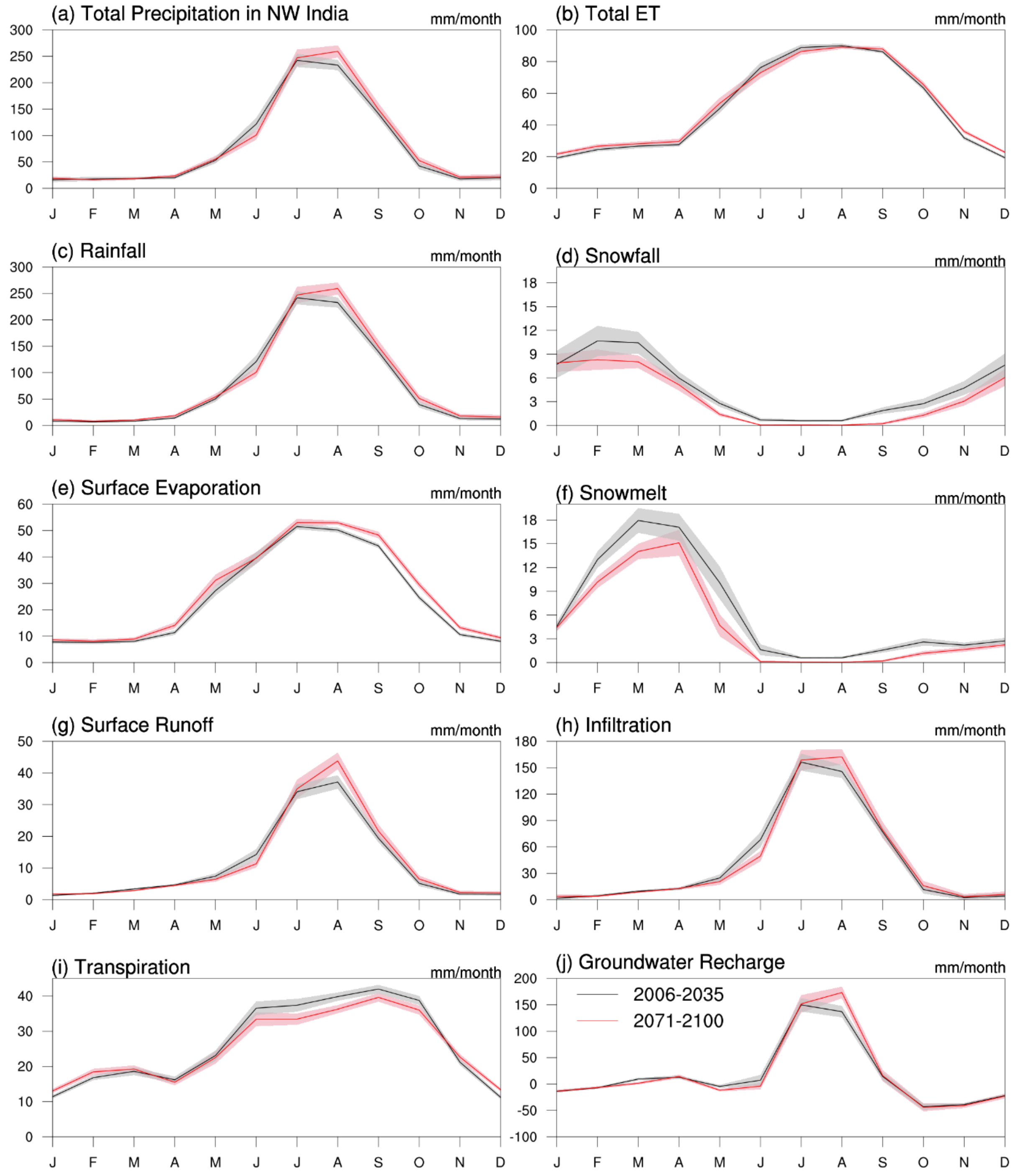

Supplementary Figure 6 | Seasonal cycle of variables over Northwestern India. (a)-(j) Black and red lines represent the ensemble mean during 2006−2035 and 2071−2100, respectively. Shaded areas represent a single standard deviation.

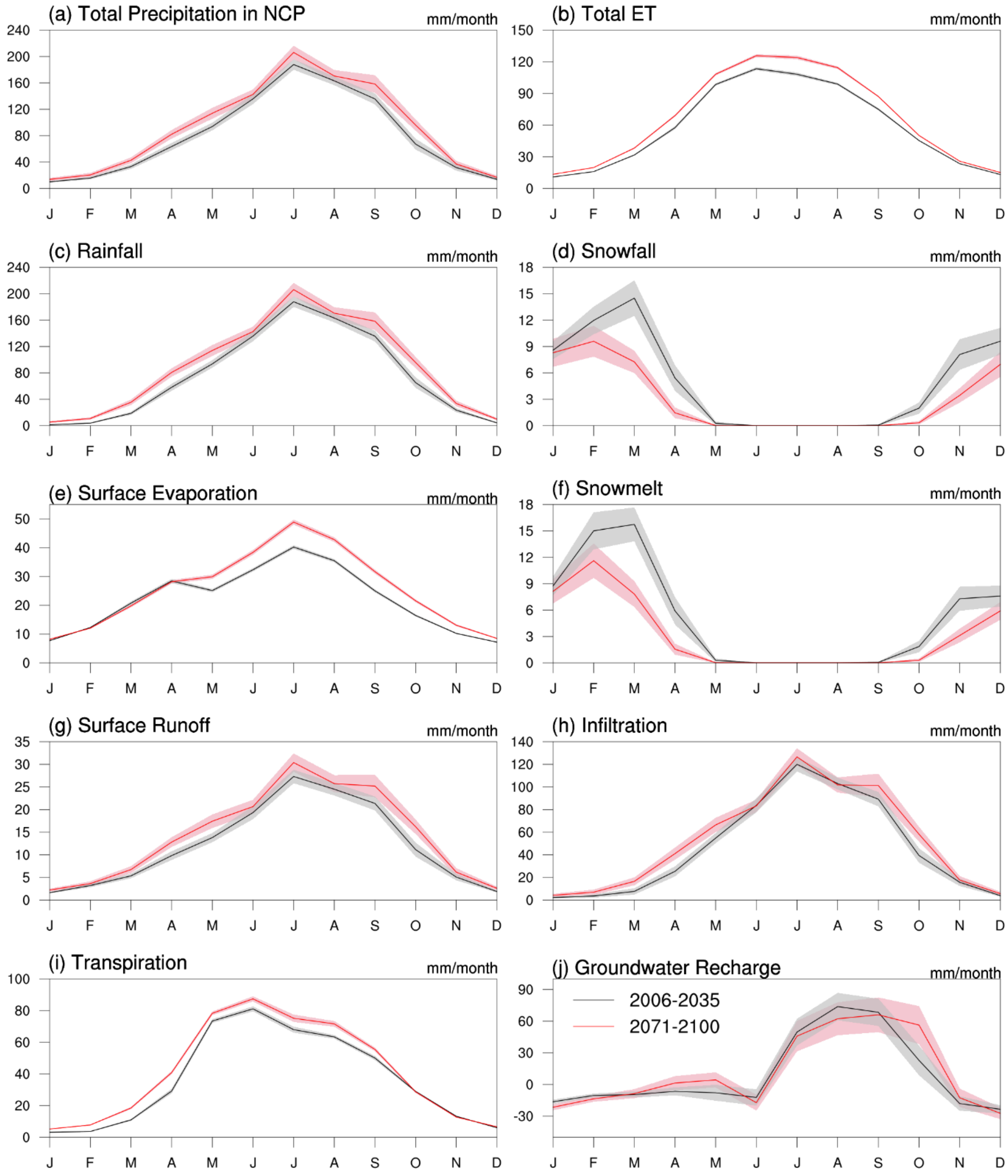

Supplementary Figure 7 | Seasonal cycle of variables over North China Plain. (a)-(j) Black and red lines represent the ensemble mean during 2006−2035 and 2071−2100, respectively. Shaded areas represent a single standard deviation.

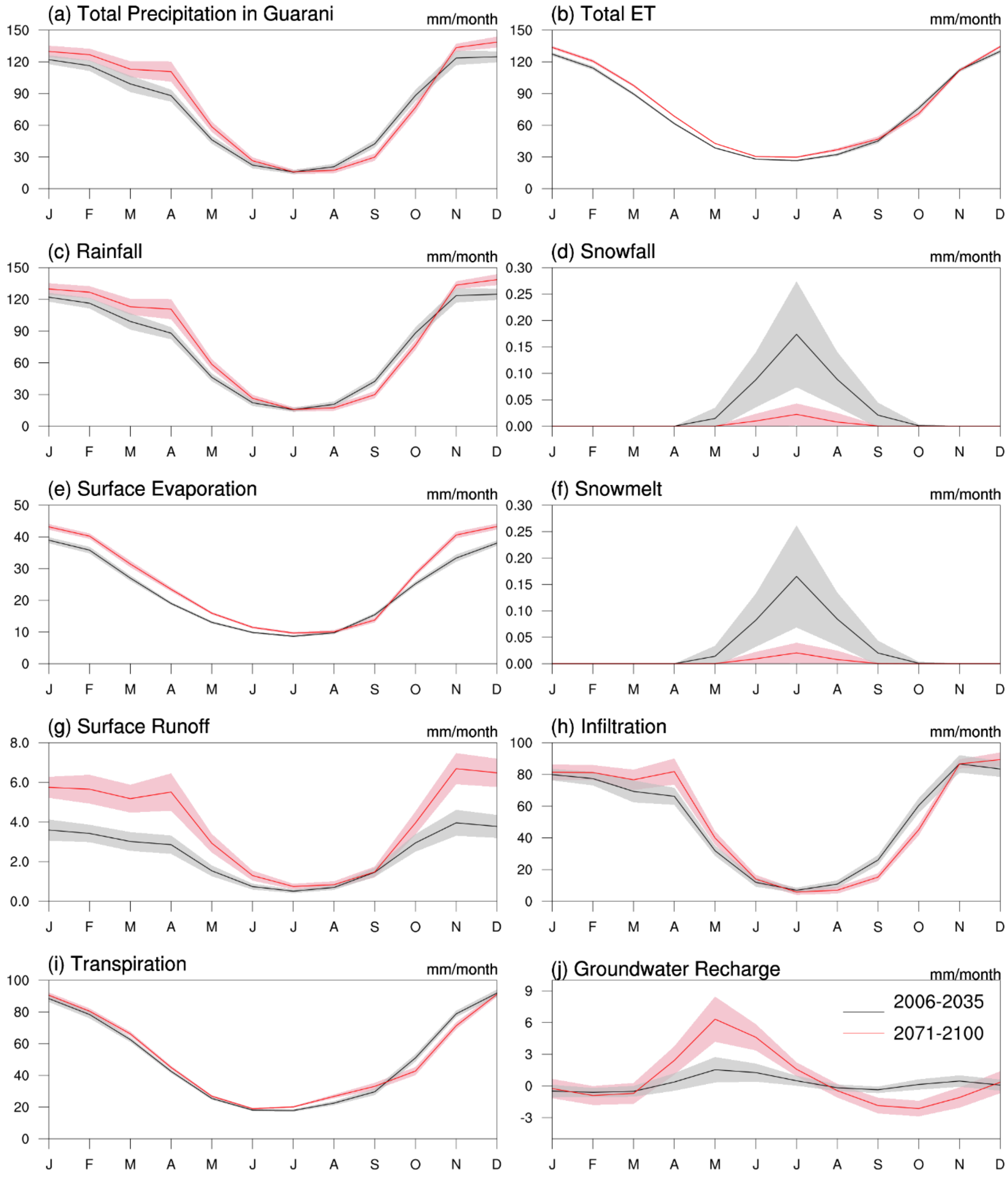

Supplementary Figure 8 | Seasonal cycle of variables over Guarani. (a)-(j) Black and red lines represent the ensemble mean during 2006−2035 and 2071−2100, respectively. Shaded areas represent a single standard deviation.

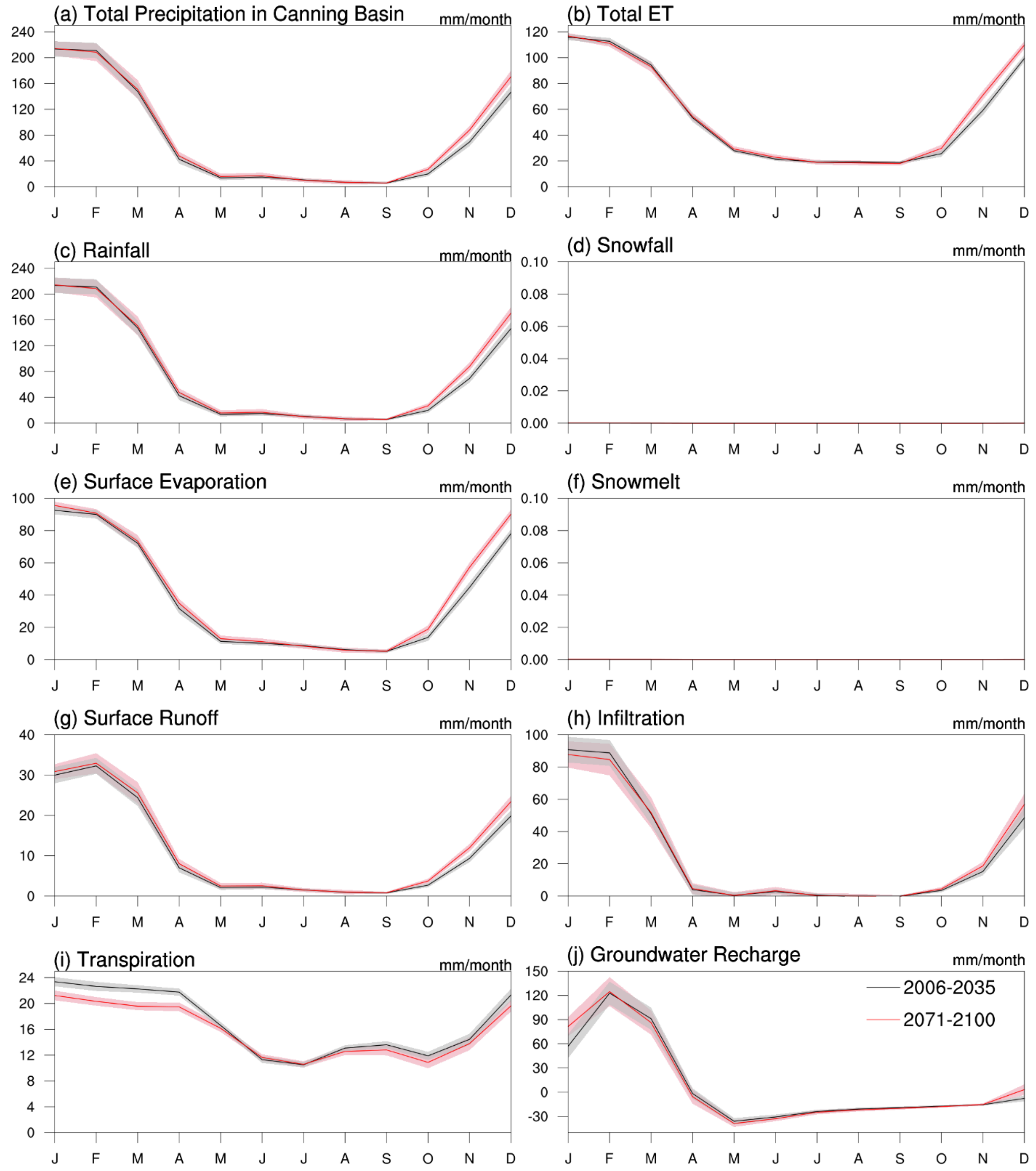

Supplementary Figure 9 | Seasonal cycle of variables over the Canning Basin. (a)-(j) Black and red lines represent the ensemble mean during 2006−2035 and 2071−2100, respectively. Shaded areas represent a single standard deviation.

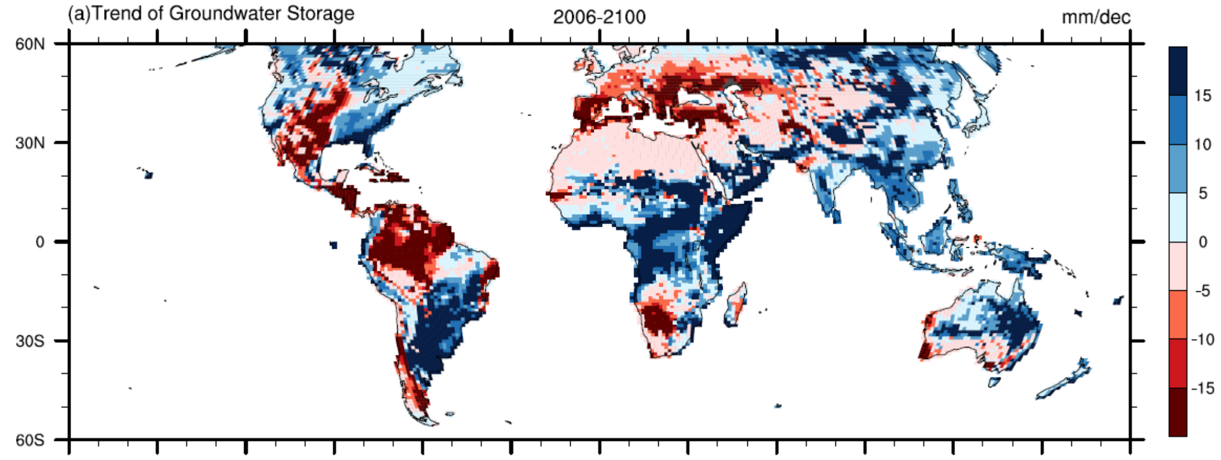

(b)

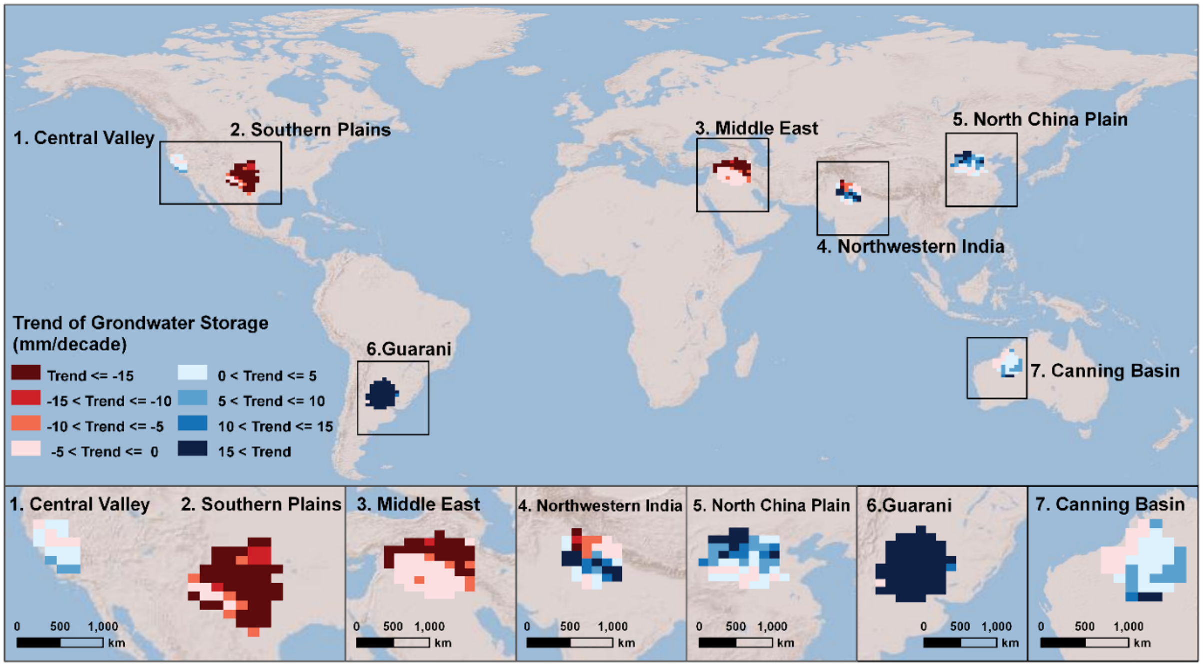

Supplementary Figure 10 | Same as Figure 2a in the main text, but for with spatial heterogeneity of original model resolution (a) a global extent with original model resolution (b) masked for studied region

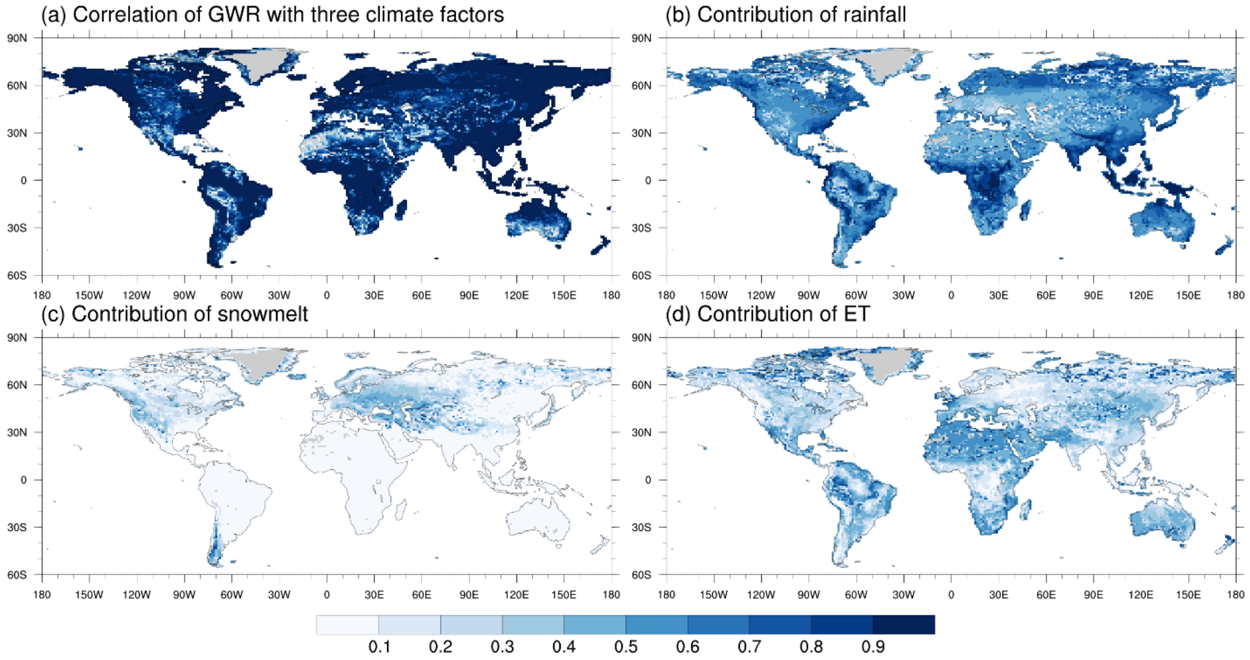

Supplementary Figure 11 | Details of Figure 3 in the main text (a) R^2 of the regression, (b-d) Contribution of each factor (rainfall, snowmelt, ET) to the groundwater recharge

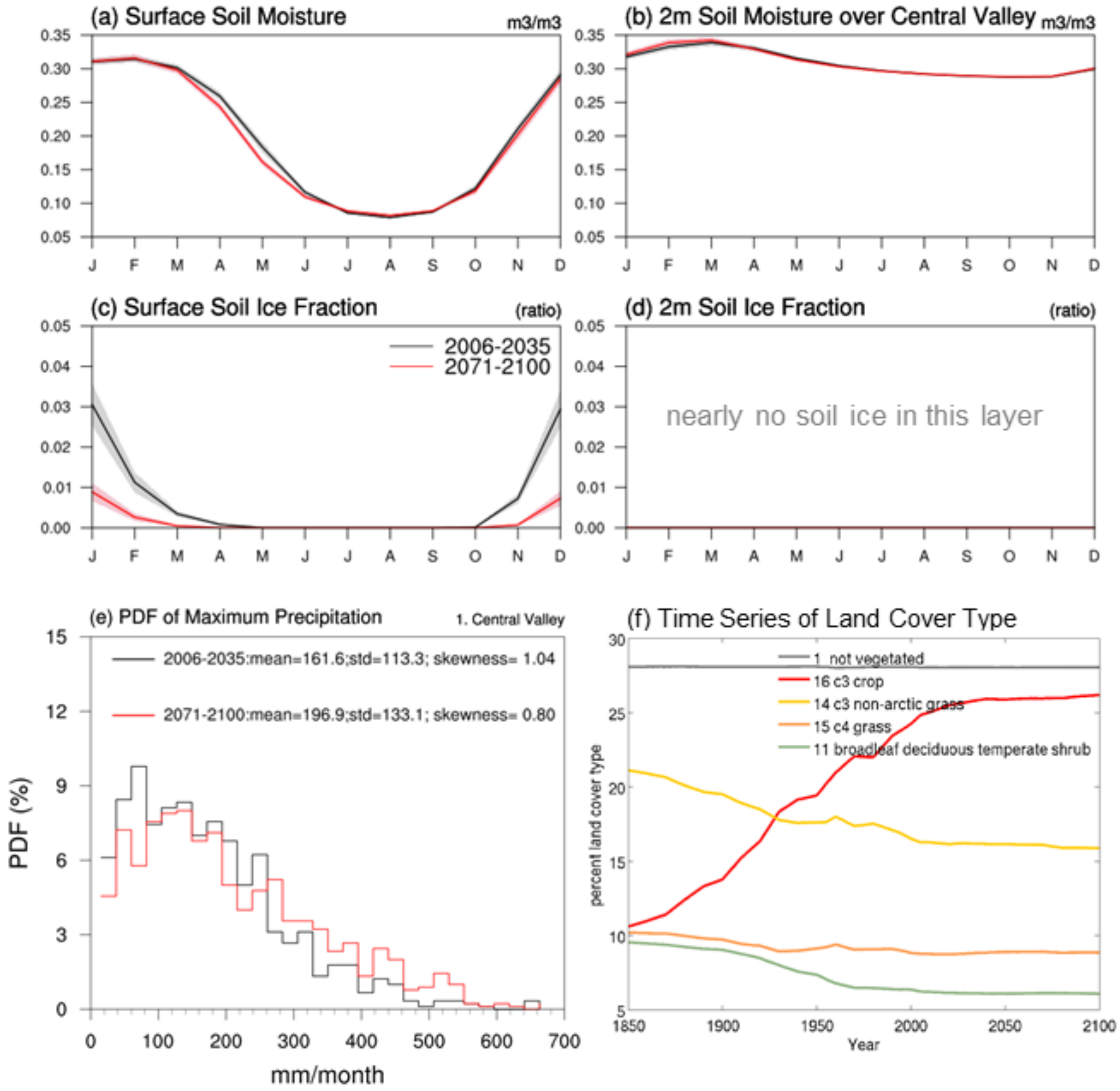

Supplementary Figure 12 | (a)- (d) Seasonal cycle of variables over the Central Valley. Black and red lines represent the ensemble mean/individual ensemble members during 2006−2035 and 2071−2100, respectively. Shaded areas represent a single standard deviation. (e) Probability distribution of maximum monthly precipitation (f) Time series of transient land cover types in CLM.

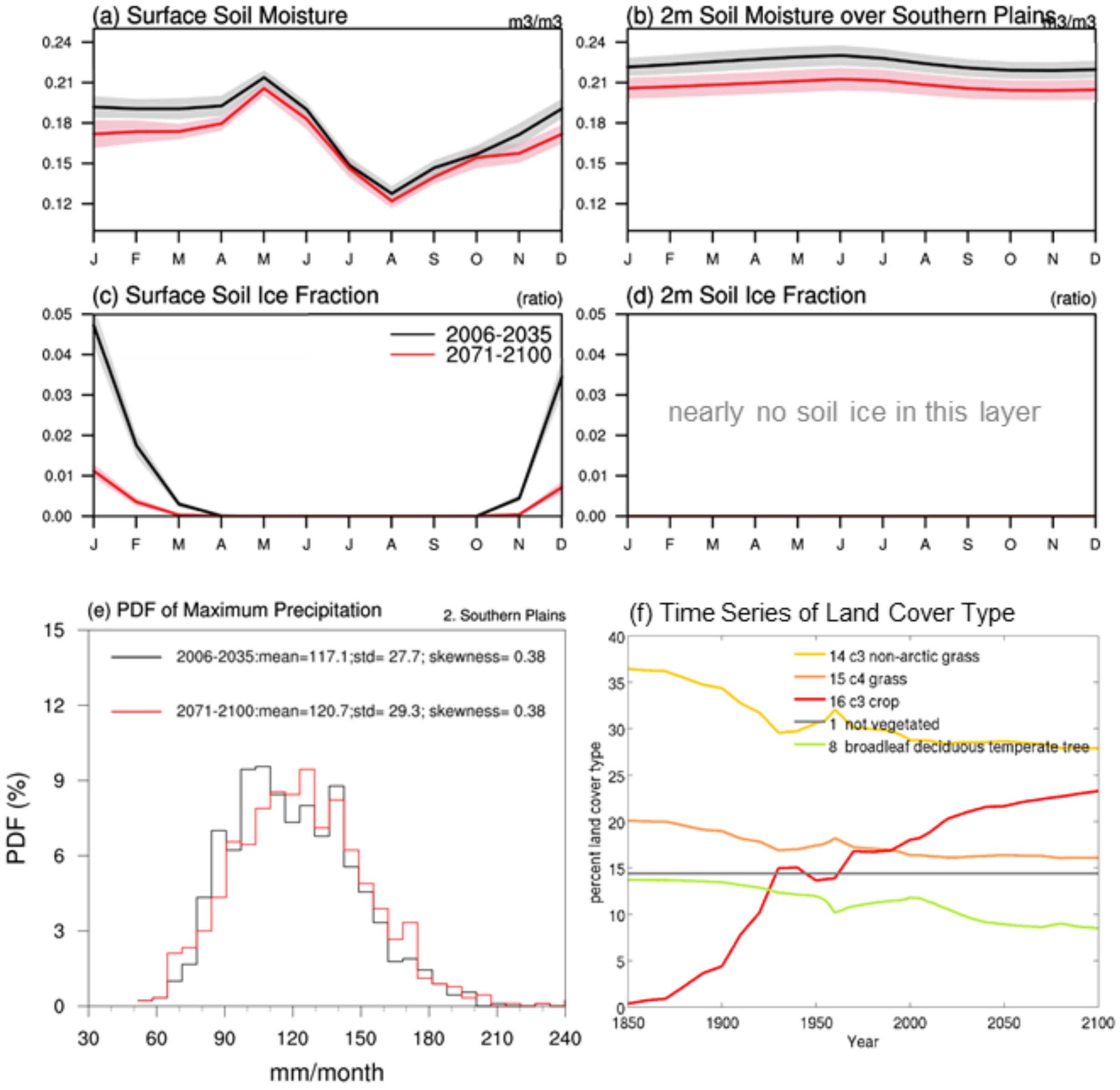

Supplementary Figure 13 | (a)- (d) Seasonal cycle of variables over the Southern Plains. Black and red lines represent the ensemble mean/individual ensemble members during 2006−2035 and 2071−2100, respectively. Shaded areas represent a single standard deviation. (e) Probability distribution of maximum monthly precipitation (f) Time series of transient land cover types in CLM.

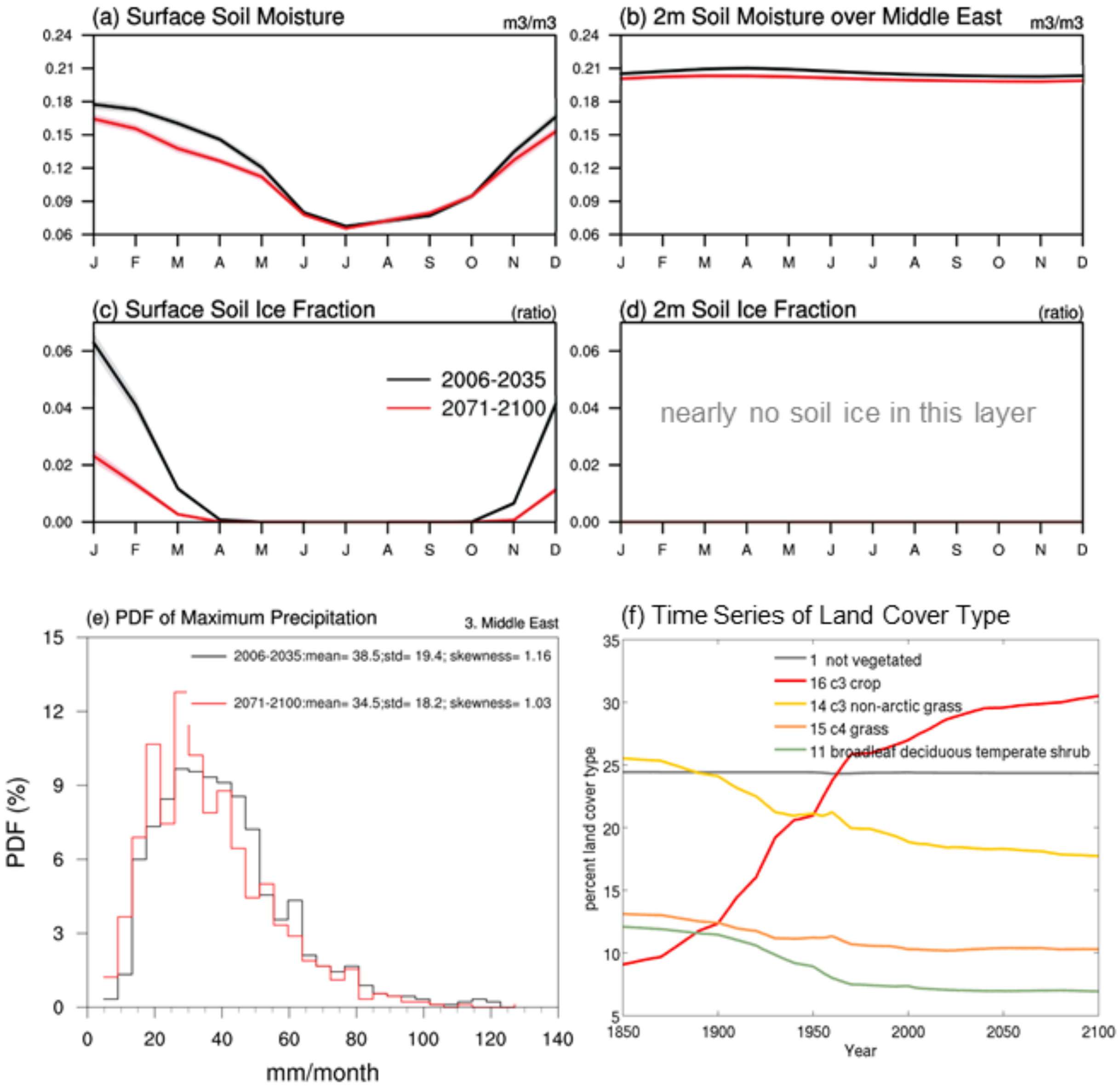

Supplementary Figure 14 | (a)- (d) Seasonal cycle of variables over Middle East. Black and red lines represent the ensemble mean/individual ensemble members during 2006−2035 and 2071−2100, respectively. Shaded areas represent a single standard deviation. (e) Probability distribution of maximum monthly precipitation (f) Time series of transient land cover types in CLM.

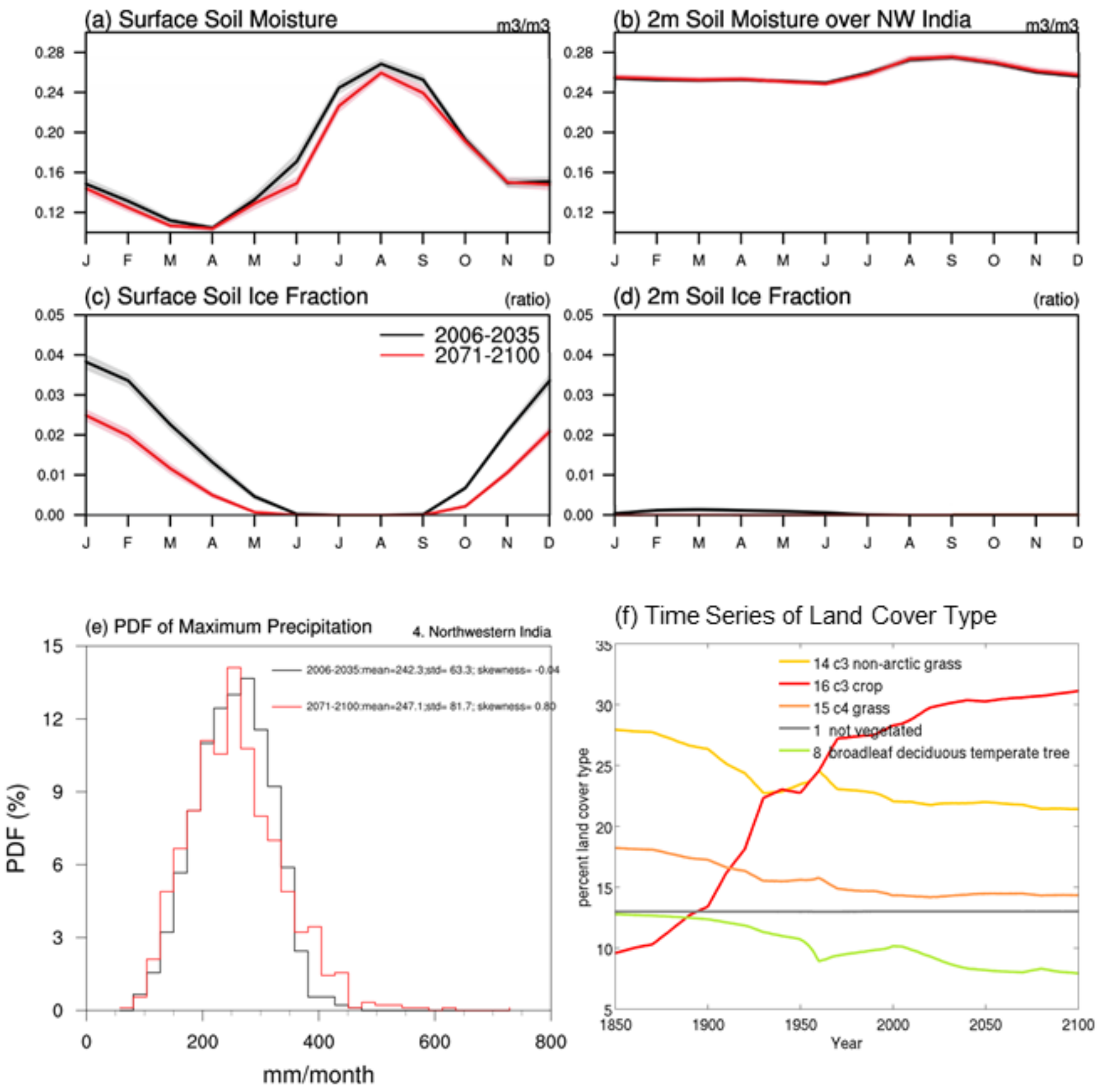

Supplementary Figure 15 | (a)- (d) Seasonal cycle of variables over the Northwestern India. Black and red lines represent the ensemble mean/individual ensemble members during 2006−2035 and 2071−2100, respectively. Shaded areas represent a single standard deviation. (e) Probability distribution of maximum monthly precipitation (f) Time series of transient land cover types in CLM.

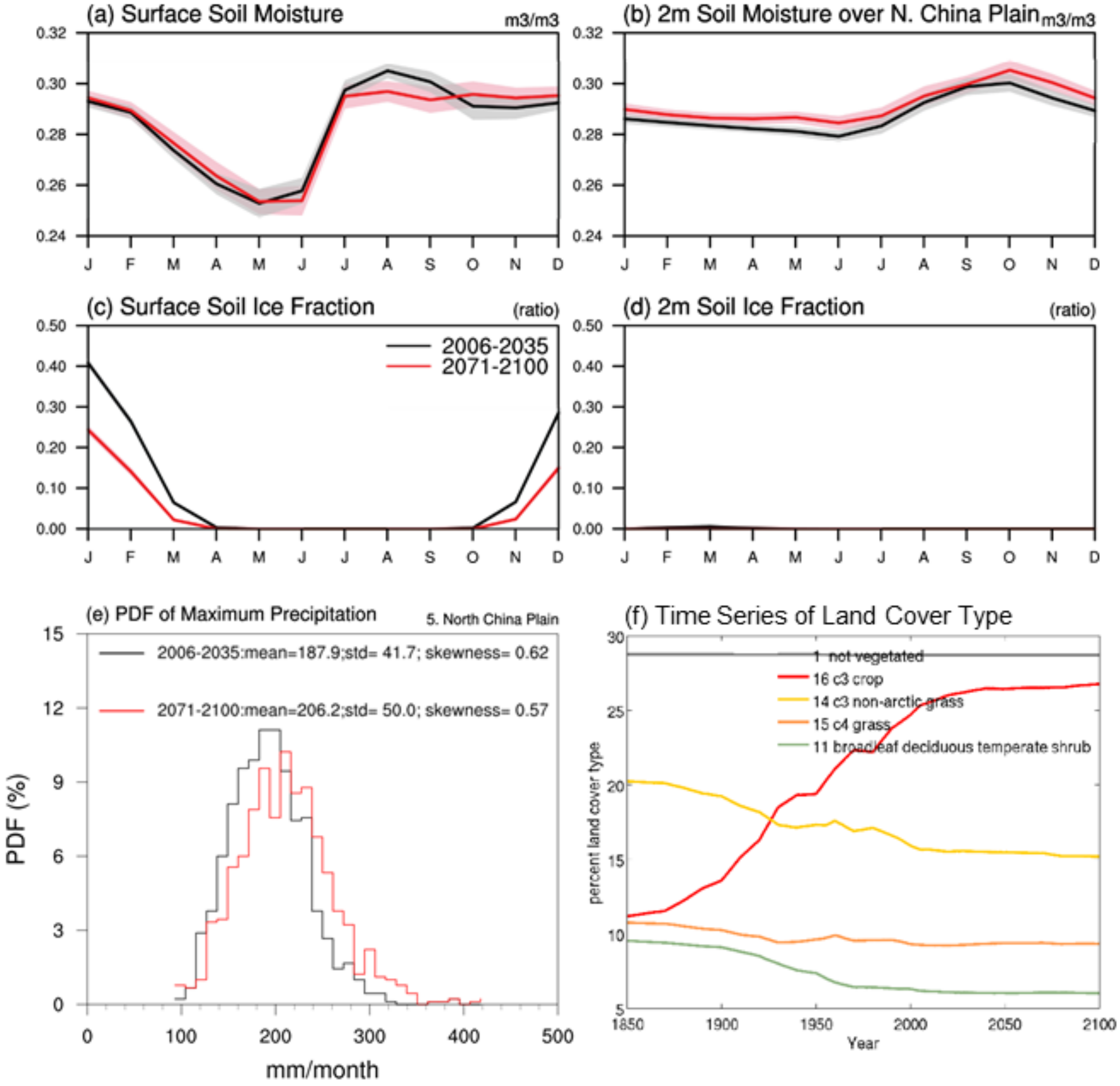

Supplementary Figure 16 | (a)- (d) Seasonal cycle of variables over North China Plains. Black and red lines represent the ensemble mean/individual ensemble members during 2006−2035 and 2071−2100, respectively. Shaded areas represent a single standard deviation. (e) Probability distribution of maximum monthly precipitation (f) Time series of transient land cover types in CLM.

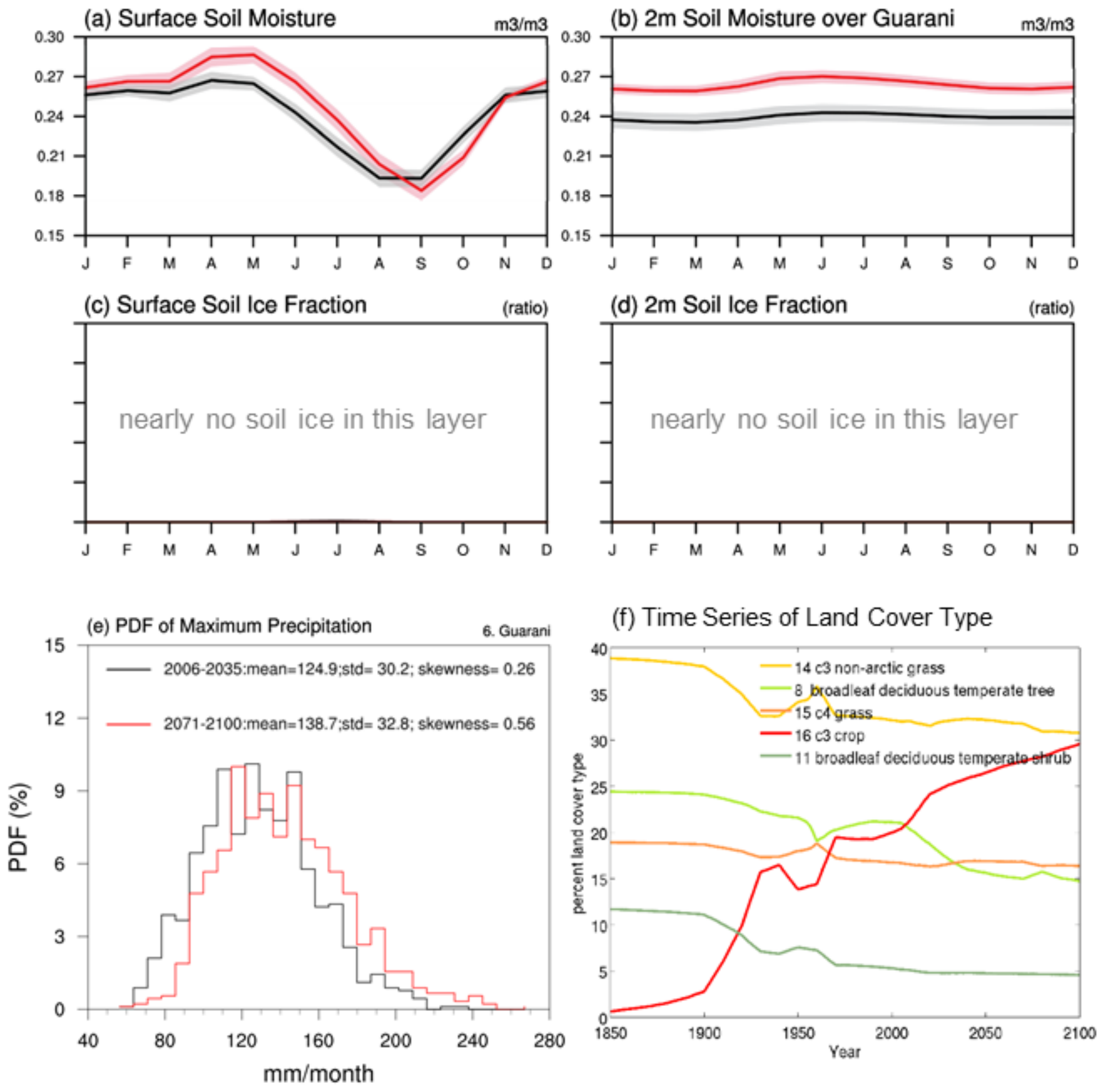

Supplementary Figure 17 | (a)- (d) Seasonal cycle of variables over the Guarani. Black and red lines represent the ensemble mean/individual ensemble members during 2006−2035 and 2071−2100, respectively. Shaded areas represent a single standard deviation. (e) Probability distribution of maximum monthly precipitation (f) Time series of transient land cover types in CLM.

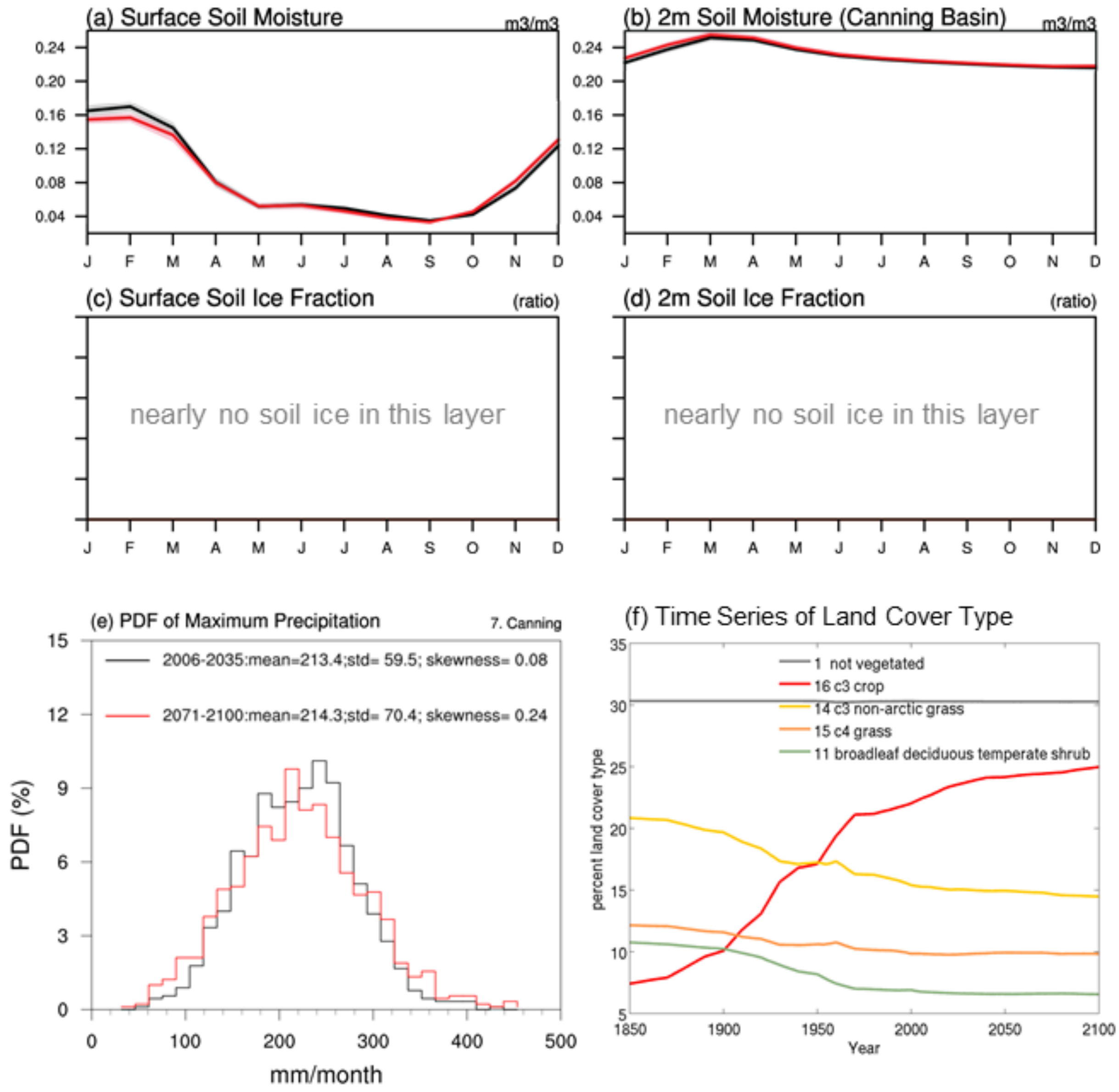

Supplementary Figure 18 | (a)- (d) Seasonal cycle of variables over Canning Basin. Black and red lines represent the ensemble mean/individual ensemble members during 2006−2035 and 2071−2100, respectively. Shaded areas represent a single standard deviation. (e) Probability distribution of maximum monthly precipitation (f) Time series of transient land cover types in CLM.

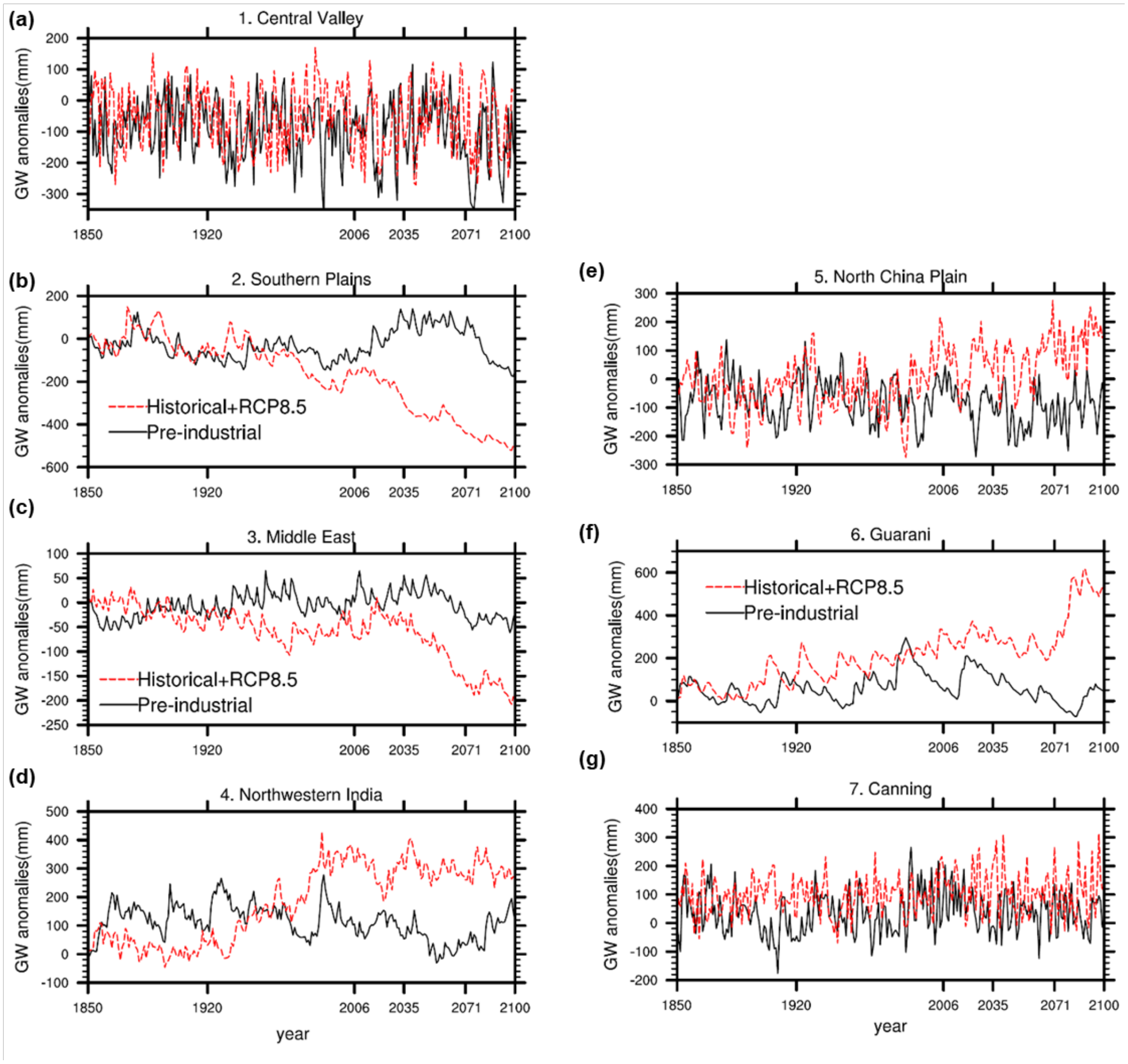

Supplementary Figure 19 | (a)-(f) 250-yr annual time series of groundwater storage under emission forcing of pre-industrial (black), and historical+RCP8.5 (red) in each aquifer

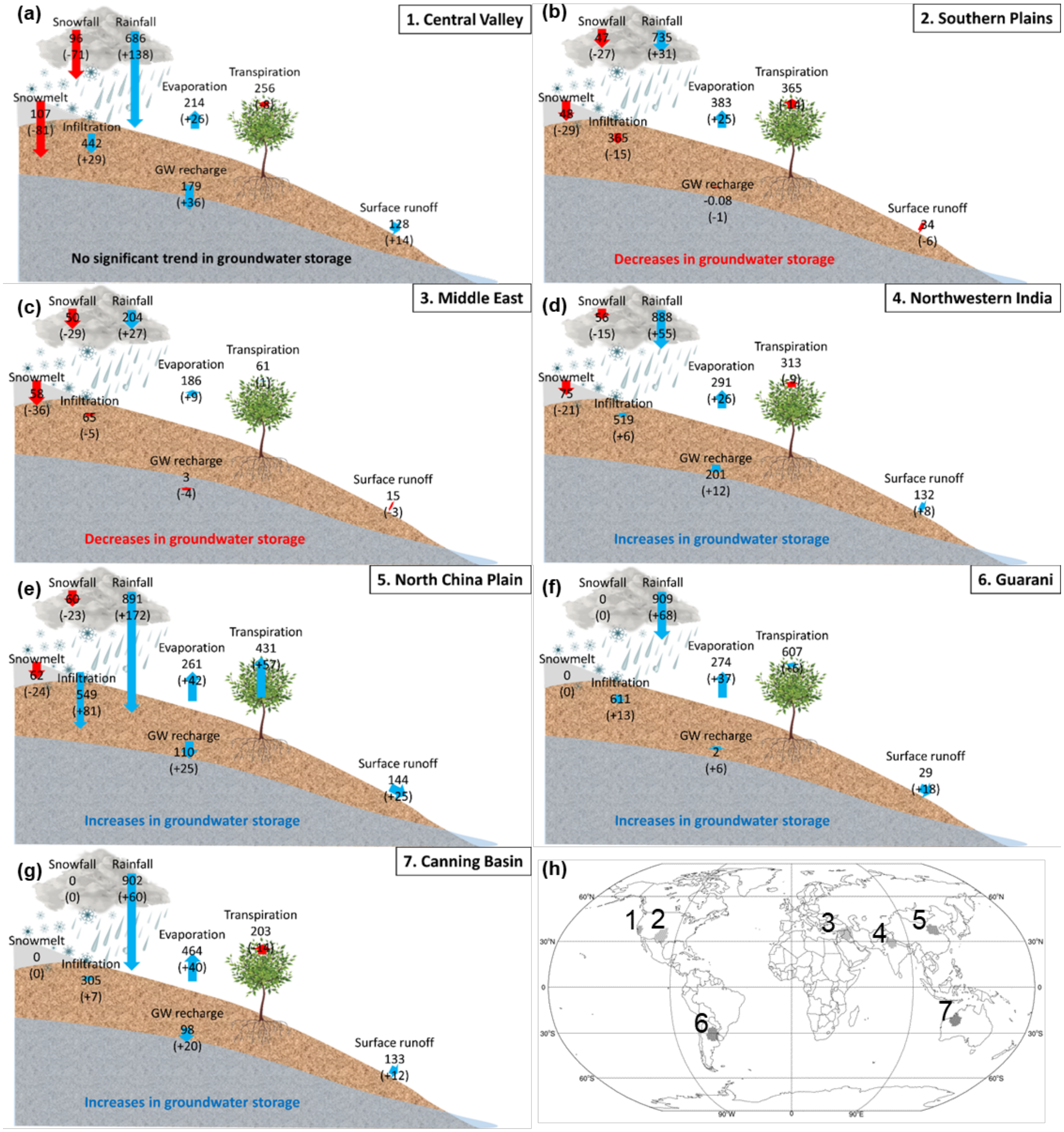

Supplementary Figure 20 | Water budget and its changes. (a)-(g) The number is the water fluxes (mm yr^{-1}) in the current water budget, while the numbers in brackets are corresponding between two periods (2071−2100 minus 2006−2035). The sizes of the arrows are proportional to the changes (number in brackets) and blue/red for increase/decrease. The direction of the arrow represents the directions of the physical fluxes (e.g., evaporation from land to the atmosphere). (h) Locations of each aquifer in (a)-(g). This figure has been designed using resources from Freepik.com.

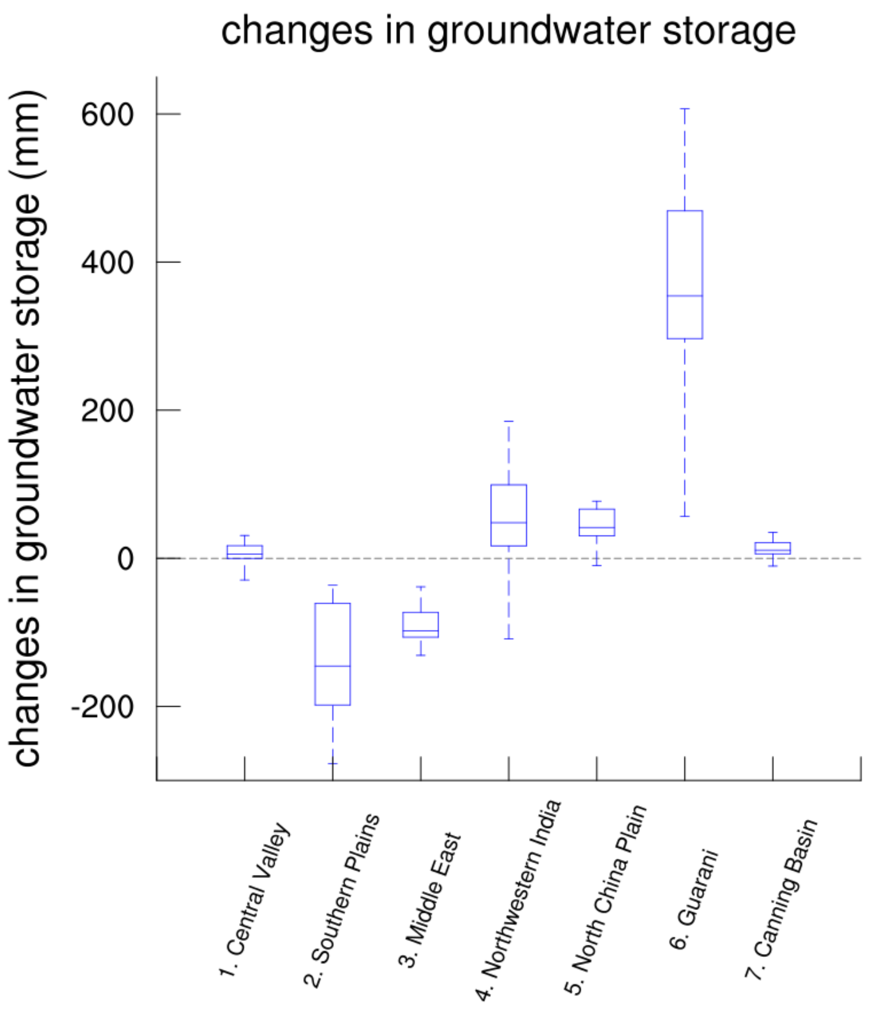

Supplementary Figure 21 | Statistics for 30 ensemble members (min, 25th percentile, median, 75th percentile, max) in each basin. Changes in groundwater in each individual ensemble member between 2071−2100 and 2006−2035

Supplementary Table 1 | Information for study region from CESM-LE

Characteristics/ Parameters	Unit	1 Central Valley	2 Southern Plains	3 Middle East	4 Northwestern India	5 North China Plain	6 Guarani	7 Canning Basin
Area	km^2	206950	677732	725055	457693	565411	701850	582624
Number of grid cell in model	-	18	56	61	36	49	56	43
Average elevation	m	898	894	792	710	837	137	325
Highest elevation	m	1716	1866	1895	5396	1528	663	515
Fraction of vegetated area	%	85	72	47	91	80	99	44
Fraction of sand at the surface layer of soil	%	48	55	45	49	44	41	76
Fraction of clay at the surface layer of soil	%	24	18	20	19	27	28	9

Supplementary Table 2 | Same as Table 1 in the main text. but for 20th century climate-driven+anthropogenic

Variable	Unit	1<br>Central Valley	2<br>Southern Plains	3<br>Middle East	4<br>Northwestern India	5<br>North China Plain	6<br>Guarani	7<br>Canning Basin
Total Precipitation	mm yr^{-1} dec^{-1}	2.5	0.4	-4.4	1.0	-5.5	1.1	12.7
Rainfall	mm yr^{-1} dec^{-1}	3.9	1.0	-3.4	0.2	-3.5	1.1	12.7
Snowfall	mm yr^{-1} dec^{-1}	-1.4	-0.6	-1.0	0.8	-2.0	0	0
Evaporation	mm yr^{-1} dec^{-1}	4.0	-1.4	-1.6	3.7	-1.7	0.6	3.3
Snowmelt	mm yr^{-1} dec^{-1}	4.1	4.8	-1.2	7.4	-0.1	0.9	6.5
Surface Runoff	mm yr^{-1} dec^{-1}	0.6	-1.4	-0.4	-0.6	-0.5	-0.4	2.9
Infiltration	mm yr^{-1} dec^{-1}	4.1	4.8	-1.2	7.4	-0.1	0.9	6.5
Transpiration	mm yr^{-1} dec^{-1}	0.4	4.1	-0.6	6.0	-1.1	-0.2	1.0
Groundwater Recharge	mm yr^{-1} dec^{-1}	4.4	1.0	-0.3	3.4	2.6	0.3	6.0
Groundwater storage	mm dec^{-1}	-13.9	-16.8	-35.5	-63.8	-5.4	-14.9	7.6

Supplementary Table 3 | Same as Table 1. but for 20th century climate-driven (CESM-001)

Variable	Unit	1<br>Central Valley	2<br>Southern Plains	3<br>Middle East	4<br>Northwestern India	5<br>North China Plain	6<br>Guarani	7<br>Canning Basin
Total Precipitation	mm yr^{-1} dec^{-1}	8.2	5.9	2.0	15.1	-4.7	1.1	-3.9
Rainfall	mm yr^{-1} dec^{-1}	6.3	6.7	1.1	14.9	-5.2	1.1	-3.9
Snowfall	mm yr^{-1} dec^{-1}	1.8	-0.9	0.9	0.2	0.6	0.1	0.0
Evaporation	mm yr^{-1} dec^{-1}	-0.6	-1.3	0.2	1.6	-3.3	-0.7	-1.3
Snowmelt	mm yr^{-1} dec^{-1}	2.0	-1.4	1.0	0.6	0.8	0.1	0.0
Surface Runoff	mm yr^{-1} dec^{-1}	1.9	-1.0	0.1	3.7	-0.4	0.1	-0.7
Infiltration	mm yr^{-1} dec^{-1}	7.0	8.1	1.6	9.9	-1.0	1.7	-2.0
Transpiration	mm yr^{-1} dec^{-1}	1.1	8.4	1.0	3.7	-2.2	1.5	-1.1
Groundwater Recharge	mm yr^{-1} dec^{-1}	6.5	-0.7	0.6	5.8	1.7	-0.1	-1.1
Groundwater storage	mm dec^{-1}	4.5	-18.4	-3.8	37.7	-3.2	11.7	-2.2

Supplementary Reference

1 Taylor, K. E., Stouffer, R. J. & Meehl, G. A. An overview of CMIP5 and the experiment design. *Bulletin of the American Meteorological Society* **93**, 485-498 (2012).

2 Taylor, R. G. *et al.* Evidence of the dependence of groundwater resources on extreme rainfall in East Africa. *Nature Clim. Change* **3**, 374-378 (2013).

3 Yang, Y., Roderick, M. L., Zhang, S., McVicar, T. R. & Donohue, R. J. Hydrologic implications of vegetation response to elevated CO2 in climate projections. *Nature Climate Change* **9**, 44-48, doi:10.1038/s41558-018-0361-0 (2019).

4 Green, J. K. *et al.* Large influence of soil moisture on long-term terrestrial carbon uptake. *Nature* **565**, 476-479, doi:10.1038/s41586-018-0848-x (2019).

5 Milly, P. C. D. & Dunne, K. A. Potential evapotranspiration and continental drying. *Nature Climate Change* **6**, 946, doi:10.1038/nclimate3046 (2016).

6 Hetherington, A. M. & Woodward, F. I. The role of stomata in sensing and driving environmental change. *Nature* **424**, 901, doi:10.1038/nature01843 (2003).

7 Lawrence, D. M., Thornton, P. E., Oleson, K. W. & Bonan, G. B. The Partitioning of Evapotranspiration into Transpiration, Soil Evaporation, and Canopy Evaporation in a GCM: Impacts on Land–Atmosphere Interaction. *Journal of Hydrometeorology* **8**, 862-880, doi:10.1175/jhm596.1 (2007).

8 Wada, Y. *et al.* Global depletion of groundwater resources. *Geophysical Research Letters* **37**, doi:10.1029/2010GL044571 (2010).

9 Wada, Y. *et al.* Fate of water pumped from underground and contributions to sea-level rise. *Nature Climate Change* **6**, 777(2016).

10 Döll, P. Vulnerability to the impact of climate change on renewable groundwater resources: a global-scale assessment. Environmental Research Letters 4, 035006 (2009).

11 Portmann, F., Petra, D., Stephanie, E. & Martina, F. Impact of climate change on renewable groundwater resources: assessing the benefits of avoided greenhouse gas emissions using selected CMIP5 climate projections. Environmental Research Letters 8, 024023 (2013).

12 Meixner, T. et al. Implications of projected climate change for groundwater recharge in the western United States. Journal of Hydrology 534, 124-138 (2016).
