# Supplementary material for: Divergent effects of climate change on future groundwater availability in key mid-latitude aquifers
Source: Nat Commun. 2020 Jul 24;11:3710. doi: 10.1038/s41467-020-17581-y (PMC7382464; doi:10.1038/s41467-020-17581-y)
Supplement: Supplementary file 3 — Reporting Summary [file 41467_2020_17581_MOESM3_ESM.pdf]

nature research

Corresponding author(s): Min-Hui Lo

Last updated by author(s): Jun 1, 2020

Reporting Summary

Nature Research wishes to improve the reproducibility of the work that we publish. This form provides structure for consistency and transparency in reporting. For further information on Nature Research policies, see Authors & Referees and the Editorial Policy Checklist .

Statistics

For all statistical analyses, confirm that the following items are present in the figure legend, table legend, main text, or Methods section.

n/a	Confirmed	
☐	☒	The exact sample size (*n*) for each experimental group/condition, given as a discrete number and unit of measurement
☒	☐	A statement on whether measurements were taken from distinct samples or whether the same sample was measured repeatedly
☐	☒	The statistical test(s) used AND whether they are one- or two-sided<br>*Only common tests should be described solely by name; describe more complex techniques in the Methods section.*
☐	☒	A description of all covariates tested
☐	☒	A description of any assumptions or corrections, such as tests of normality and adjustment for multiple comparisons
☐	☒	A full description of the statistical parameters including central tendency (e.g. means) or other basic estimates (e.g. regression coefficient) AND variation (e.g. standard deviation) or associated estimates of uncertainty (e.g. confidence intervals)
☐	☒	For null hypothesis testing, the test statistic (e.g. *F*, *t*, *r*) with confidence intervals, effect sizes, degrees of freedom and *P* value noted<br>*Give P values as exact values whenever suitable.*
☒	☐	For Bayesian analysis, information on the choice of priors and Markov chain Monte Carlo settings
☐	☒	For hierarchical and complex designs, identification of the appropriate level for tests and full reporting of outcomes
☐	☒	Estimates of effect sizes (e.g. Cohen's *d*, Pearson's *r*), indicating how they were calculated

Our web collection on statistics for biologists contains articles on many of the points above.

Software and code

Policy information about availability of computer code

Data collection	All data used for analysis are publicly available or open access. See Data Section for more detail.
Data analysis	Codes for analysis and visualization are written in NCAR Command Language (NCL) and MATLAB.

For manuscripts utilizing custom algorithms or software that are central to the research but not yet described in published literature, software must be made available to editors/reviewers. We strongly encourage code deposition in a community repository (e.g. GitHub). See the Nature Research guidelines for submitting code & software for further information.

Data

Policy information about availability of data

All manuscripts must include a data availability statement. This statement should provide the following information, where applicable:

- Accession codes, unique identifiers, or web links for publicly available datasets
- A list of figures that have associated raw data
- A description of any restrictions on data availability

CESM is a community ESM. The model code for CESM is released http://www.cesm.ucar.edu/models/. CESM Large Ensemble Community Project generated by NCAR is available http://www.cesm.ucar.edu/projects/community-projects/LENS/. GRACE land data are available at http://grace.jpl.nasa.gov, supported by the NASA MEaSUREs Program. The GLDAS data used in this study were acquired as part of the mission of NASA's Earth Science Division and archived and distributed by the Goddard Earth Sciences (GES) Data and Information Services Center (DISC). Our co-author provided simulations from Wada et al. (2016).

Field-specific reporting

Please select the one below that is the best fit for your research. If you are not sure, read the appropriate sections before making your selection.

October 2018

Ecological, evolutionary & environmental sciences study design

All studies must disclose on these points even when the disclosure is negative.

Study description	Our study is based on remote sensing datasets and model outputs.
Research sample	All available data (all ensemble members, all grids inside aquifers) is used.
Sampling strategy	All simulations are used for statistical analysis. No artificial-filtered is used. Sample sizes for regression are more than 80.
Data collection	Data used are directly downloaded to Linux machine with shell scripts. All data are accessible.
Timing and spatial scale	Data are downloaded in 2015 -2019. The data describe the Earth System from 1850-2100.
Data exclusions	No data are excluded without description.
Reproducibility	All analysis and visualization are reproducible.
Randomization	No randomization is applied in our analysis.
Blinding	Blinding is not relevant to climate modeling.

Did the study involve field work? ☐ Yes ☒ No

Reporting for specific materials, systems and methods

We require information from authors about some types of materials, experimental systems and methods used in many studies. Here, indicate whether each material, system or method listed is relevant to your study. If you are not sure if a list item applies to your research, read the appropriate section before selecting a response.

Materials & experimental systems

n/a	Involved in the study	
☒	☐	Antibodies
☒	☐	Eukaryotic cell lines
☒	☐	Palaeontology
☒	☐	Animals and other organisms
☒	☐	Human research participants
☒	☐	Clinical data

Methods

n/a	Involved in the study	
☒	☐	ChIP-seq
☒	☐	Flow cytometry
☒	☐	MRI-based neuroimaging
